# Delayed Gastric Bleeding in a Patient With Chronic Myeloid Leukemia: A Case of Post‐Biopsy Bleeding

**DOI:** 10.1002/deo2.70337

**Published:** 2026-04-29

**Authors:** Yoko Kosaka, Hiromichi Yamane, Hidekazu Nakanishi, Nagio Takigawa

**Affiliations:** ^1^ Department of General Internal Medicine 4 Kawasaki Medical School Okayama Japan

**Keywords:** chronic myeloid leukemia, complication, delayed bleeding, diagnostic esophagogastroduodenoscopy, underlying risk factor

## Abstract

A 76‐year‐old man was referred to our hospital for evaluation of anemia. Although he was elderly (≥75 years), no apparent risk factors for bleeding during endoscopic biopsy, such as renal dysfunction or anticoagulant therapy, were identified. Upper gastrointestinal endoscopy revealed extensive atrophic gastric mucosa extending from the antrum to the gastric body. A biopsy was obtained from the atrophic mucosa in the antrum for histopathological evaluation after confirming a low risk of bleeding. Eight days after the initial endoscopy, the patient was hospitalized and started on nilotinib for chronic myeloid leukemia, which had been subsequently diagnosed. Gastrointestinal bleeding occurred 5 h after the initiation of nilotinib. Emergency endoscopy revealed a reddish polypoid lesion with active bleeding at the previous biopsy site. Endoscopic polypectomy was performed, and hemostasis was achieved. Delayed gastric bleeding after upper gastrointestinal endoscopic biopsy alone is extremely rare. Current guidelines in Japan state that tissue biopsy is acceptable when clinically indicated, even in patients receiving anticoagulant or antiplatelet therapy. Although no definitive cause was identified, myeloproliferative disorders are associated with bleeding tendencies due to coagulation abnormalities and drug‐induced bleeding related to tyrosine kinase inhibitors such as dasatinib and nilotinib. These factors may have contributed to the delayed bleeding in this case. This case highlights the importance of careful follow‐up after endoscopic biopsy in patients with underlying myeloproliferative disorders.

**Trial Registration**: N/A.

## Graphical abstract

The presence of underlying conditions, particularly malignancies such as myeloproliferative disorders, may increase the risk of delayed bleeding following gastric mucosal biopsy, and similar cases warrant careful accumulation and evaluation in future.

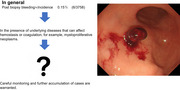

## 1 Introduction

Delayed gastric bleeding refers to hemorrhage from the stomach that occurs after a certain period of time—typically several days to approximately 2 weeks—following therapeutic procedures such as endoscopic submucosal dissection (ESD) or gastrectomy. This condition most commonly presents as an adverse event associated with ESD. In contrast, clinically significant bleeding after diagnostic upper gastrointestinal endoscopy with biopsy is thought to be rare, except in patients with specific risk factors such as advanced age, parenchymal organ dysfunction, or the use of anticoagulant therapy [1, 2]. A Japanese prospective study reported that bleeding after upper gastrointestinal endoscopic biopsy occurred in only 0.15% of cases (6/3758), indicating that clinically significant bleeding is rare in Japan [3]. From the 1990s onward, several reports have suggested that patients with myeloproliferative neoplasms (MPNs), particularly those with chronic myeloid leukemia (CML), are prone to thrombotic events and bleeding tendencies, especially gastrointestinal hemorrhage [4]. Although there is a limitation in that the underlying disease is rare, the risk of post‐biopsy bleeding in patients with MPNs should be considered an issue warranting careful evaluation. Herein, we report a case of a patient with underlying CML who developed acute anemia and upper gastrointestinal bleeding 8 days after undergoing endoscopic gastric biopsy.

## 2 Case Report

**FIGURE 1:**
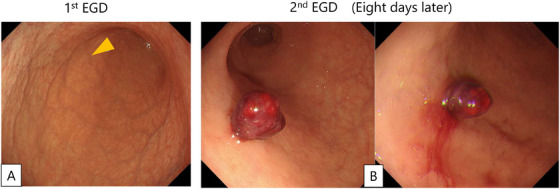
(A) Endoscopic findings revealed severe atrophic gastritis predominantly involving the gastric body. An endoscopic biopsy was obtained from the anterior wall of the antrum to exclude autoimmune gastritis (orange arrowhead). (B) On emergency esophagogastroduodenoscopy, active bleeding was observed from a polypoid lesion at the previous biopsy site, which was considered the source of hemorrhage.

A 76‐year‐old man presented with leukocytosis and anemia. A fecal occult blood test was positive, and esophagogastroduodenoscopy (EGD) was performed to evaluate suspected gastrointestinal bleeding. Endoscopic examination revealed severe atrophic gastritis predominantly involving the gastric body, and endoscopic biopsy was performed to screen for Helicobacter pylori infection, autoimmune gastritis, and other causes of atrophic gastritis (Figure 1A, orange arrowhead). Histopathological examination demonstrated atrophy of the fundic glands without evidence of enterochromaffin‐like cell hyperplasia. Autoimmune gastritis was therefore considered unlikely, and the patient was diagnosed with chronic gastritis. Laboratory testing revealed marked leukocytosis (365,670/µL) and mildly macrocytic anemia (hemoglobin 8.0 g/dL; mean corpuscular volume 102.2 fL), with a normal platelet count and normal coagulation parameters. Bone marrow aspiration showed hypercellular marrow, and cytogenetic analysis identified t(9;22), confirming chronic‐phase CML (CML‐CP). Nilotinib monotherapy was initiated 8 days after the initial EGD.

**FIGURE 2:**
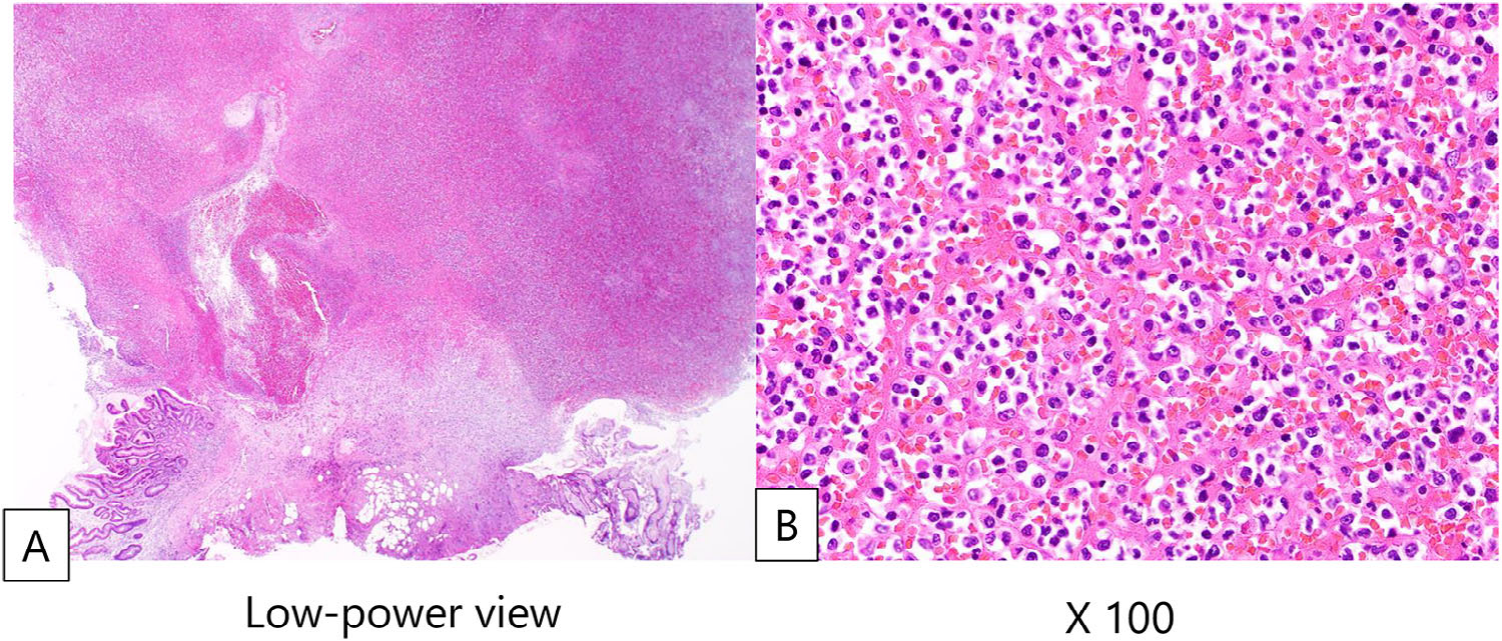
(A, B) The bleeding polypoid lesion was identified as an inflammatory granuloma with dilated capillaries as a histopathological diagnosis. No infiltration of leukemic cells was observed.

Approximately 5 h after the first dose, the patient developed massive hematochezia, and the hemoglobin level decreased from 8.0 to 6.2 g/dL. Emergent repeat EGD revealed active bleeding from a polypoid lesion at the previous biopsy site (Figure 1B). The lesion presented as a polypoid lesion and was considered to correspond to Forrest classification Ib (oozing bleeding). Therefore, endoscopic hemostasis was deemed appropriate, and polypectomy followed by clipping of the resection margin was performed. Endoscopic polypectomy followed by clip application successfully achieved hemostasis (Figures S1 and S2). Histopathological examination of the resected specimen demonstrated an inflammatory granuloma with dilated capillaries and no evidence of tumor cell infiltration, consistent with a reactive lesion (Figure 2A,B).

Nilotinib was temporarily discontinued to ensure stabilization of the bleeding. After hemostasis was confirmed by second‐look EGD, nilotinib monotherapy (600 mg/day) was resumed following a 9‐day interruption. Over the subsequent month, the white blood cell counts steadily decreased, and overall disease control improved. However, grade 3 neutropenia developed, accompanied by a fever of 38.5°C. Based on a clinical diagnosis of febrile neutropenia, supportive management, including antibiotic therapy, was administered for 4 weeks. After resolution of symptoms, nilotinib was restarted at a reduced dose of 400 mg/day. Subsequently, the major BCR/ABL mRNA level progressively declined. Approximately 1 year after initiation of therapy, complete remission of CML was achieved (Figure 2). During the 1‐year treatment period, treatment interruption and dose modification were required because of adverse events; however, CML remained well controlled, and no episodes of gastrointestinal bleeding involving either the upper or lower gastrointestinal tract were observed.

## 3 Discussion

Delayed bleeding after diagnostic biopsy during EGD is extremely rare [1, 2, 3]. In both Japan and the United States, the incidence of bleeding is generally estimated to be ≤1%, even among patients receiving antiplatelet or anticoagulant therapy [1, 2, 3]. For diagnostic endoscopy and biopsy procedures, current guidelines recommend that, when clinically indicated and after appropriate informed consent, biopsy can be performed without discontinuation of these medications [5].

In the present case, the most critical issue was the appropriate assessment of the underlying cause of delayed bleeding. This patient had virtually no established background factors associated with vascular fragility that could predispose to delayed bleeding. The only potential risk factor was advanced age (76 years). Although the underlying disease was CML‐CP and laboratory findings included anemia and leukocytosis, these conditions have not been recognized as risk factors for delayed post‐endoscopic bleeding in the existing literature.

**FIGURE 3:**
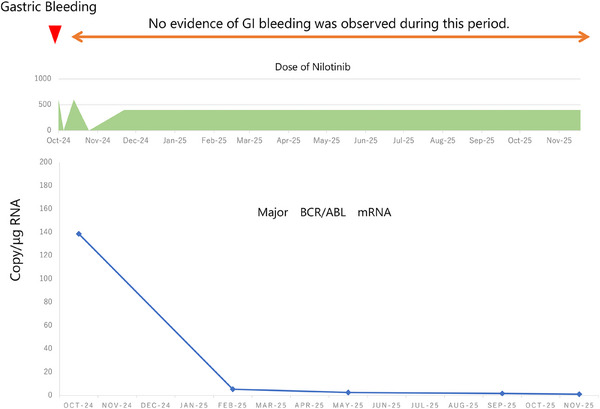
Although treatment was temporarily interrupted and the dosage was subsequently adjusted due to adverse events, control of chronic myeloid leukemia (CML) remained favorable thereafter, and no further episodes of gastrointestinal bleeding—either upper or lower—were observed.

**FIGURE 4:**
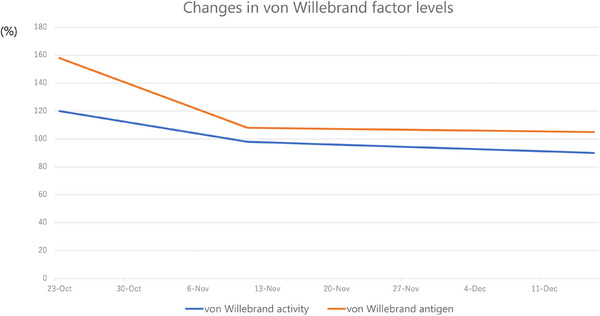
Serial measurements of von Willebrand factor performed during the early phase of the disease showed no significant decrease in von Willebrand factor levels.

Nevertheless, patients with CML have been reported to exhibit bleeding tendencies due to disease‐specific mechanisms, including acquired von Willebrand syndrome [6]. In addition, hemorrhagic gastrointestinal complications associated with tyrosine kinase inhibitors used in CML treatment have also been described [7]. These reports suggest that bleeding events in patients with CML may, at least in part, be related to the underlying disease pathophysiology. However, previously reported bleeding events have included both upper and lower gastrointestinal bleeding, limiting direct comparison with the present case. Furthermore, in our patient, no temporal decline in von Willebrand factor levels was documented (Figures 3 and 4), and no additional bleeding episodes occurred during follow‐up. Therefore, a definitive causative mechanism could not be established. Granulation tissue is characterized by abundant neovascularization and fragile capillary structures, making it prone to bleeding. Previous reports have demonstrated that lesions composed of granulation tissue, such as pyogenic granulomas and inflammatory polyps, can present with gastrointestinal bleeding and may bleed easily after endoscopic manipulation [8]. Therefore, it is plausible that even minimal mechanical stimulation, such as biopsy, may trigger bleeding in such lesions. In the present case, the coexistence of fragile granulation tissue and an underlying bleeding diathesis associated with CML may have synergistically contributed to the development of post‐biopsy bleeding.

Although delayed bleeding after gastric mucosal biopsy is extremely rare, the present case, together with previous reports demonstrating an increased epidemiological risk of bleeding, suggests that potential disease‐related bleeding tendencies in patients with MPNs may warrant careful consideration [6, 7, 9]. From this perspective, it may be worthwhile to consider the possible contribution of underlying hematologic disorders when interpreting unexpected post‐biopsy bleeding. Further accumulation of similar cases will be necessary to clarify potential risk factors and to better understand the mechanisms underlying delayed post‐biopsy hemorrhage.

## Author Contributions

**Yoko Kosaka** and **Hiromichi Yamane** planned this study. **Yoko Kosaka**, **Hidekazu Nakanishi**, and **Hiromichi Yamane** were involved in collecting clinical data from medical records. **Yoko Kosaka** and **Hiromichi Yamane** analyzed the data. **Yoko Kosaka**, **Hiromichi Yamane**, and **Nagio Takigawa** wrote this paper.

## Conflicts of Interest

The authors declare no conflicts of interest.

## Funding

The authors have nothing to report.

## Ethics Statement

**Approval of the research protocol by an Institutional Reviewer Board**: N/A.

## Consent

The written consent of the patient has been obtained.

## Supporting information




**FIGURE S1** (A) Active oozing of blood was observed from a polypoid　lesion, which was considered consistent with Forrest classification Ib. (B) Hypertonic saline epinephrine injection was administered to the base of the lesion. The base of the polyp appeared pale (discolored). (C) Polypectomy was performed; however, no bleeding point was identified on the mucosal surface. (D) Given the presence of delayed bleeding, the mucosal defect after resection was prophylactically closed using clips, and the procedure was completed.

**FIGURE S2** (A) The clips remained in place at the time of follow‐up endoscopy 1 week later. (B) An ulcer base was observed at the root of the clips; however, no bleeding was identified.
