# Supplementary figures and images for: Delayed Gastric Bleeding in a Patient With Chronic Myeloid Leukemia: A Case of Post‐Biopsy Bleeding
Source: DEN Open. 2026 Apr 29;7:e70337. doi: 10.1002/deo2.70337 (PMC13126076; doi:10.1002/deo2.70337)

## Slide 1
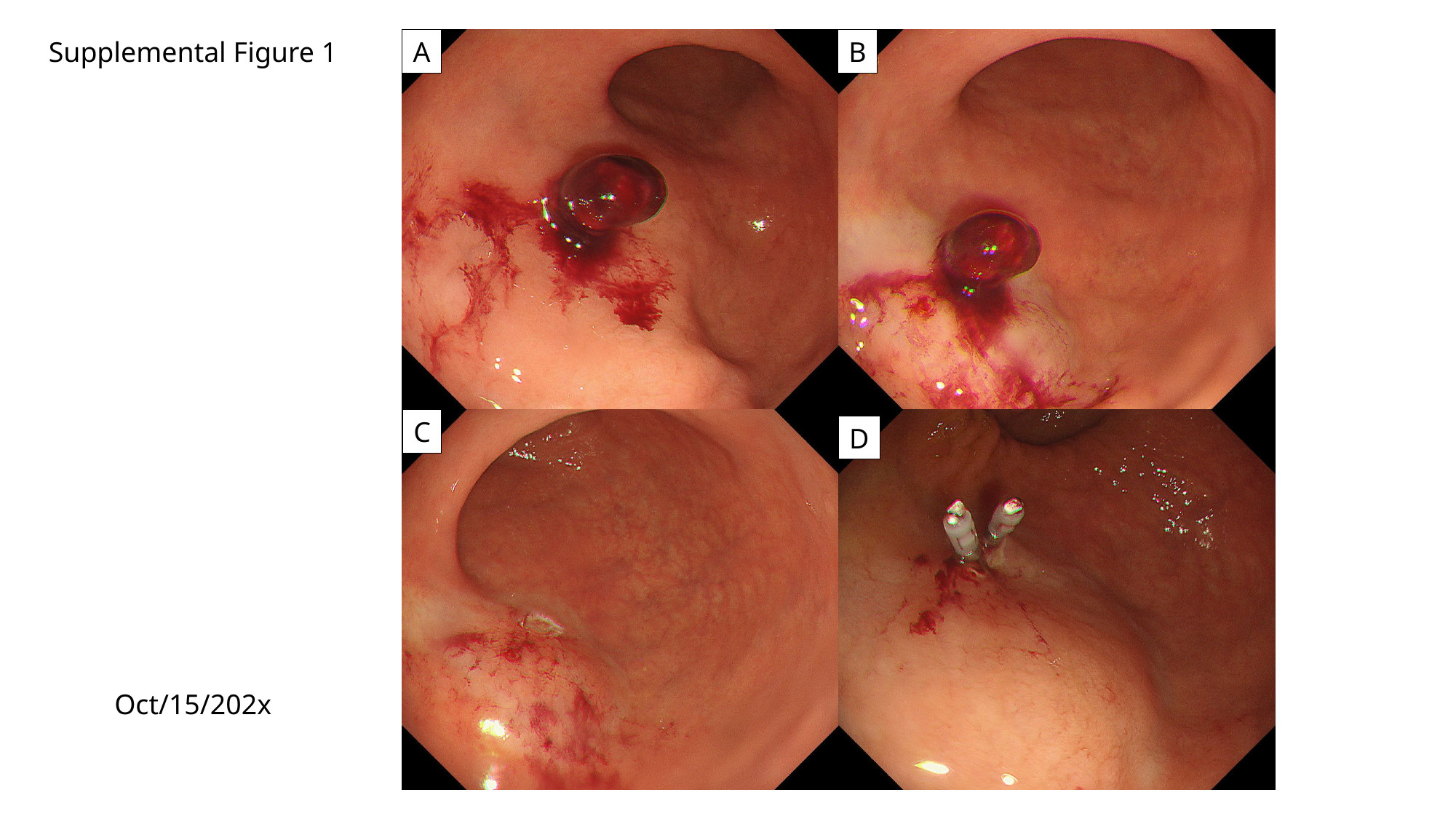

B
Supplemental Figure 1
A
C
D
Oct/15/202x

Supplement: Supplementary file 1 — FIGURE S1 (A) Active oozing of blood was observed from a polypoid lesion, which was considered consistent with Forrest classification Ib. (B) Hypertonic saline epinephrine injection was administered to the base of the lesion. The base of the polyp appeared pale (discolored). (C) Polypectomy was performed; however, no bleeding point was identified on the mucosal surface. (D) Given the presence of delayed bleeding, the mucosal defect after resection was prophylactically closed using clips, and the procedure was completed. [file DEO2-7-e70337-s002.pptx]

## Slide 1
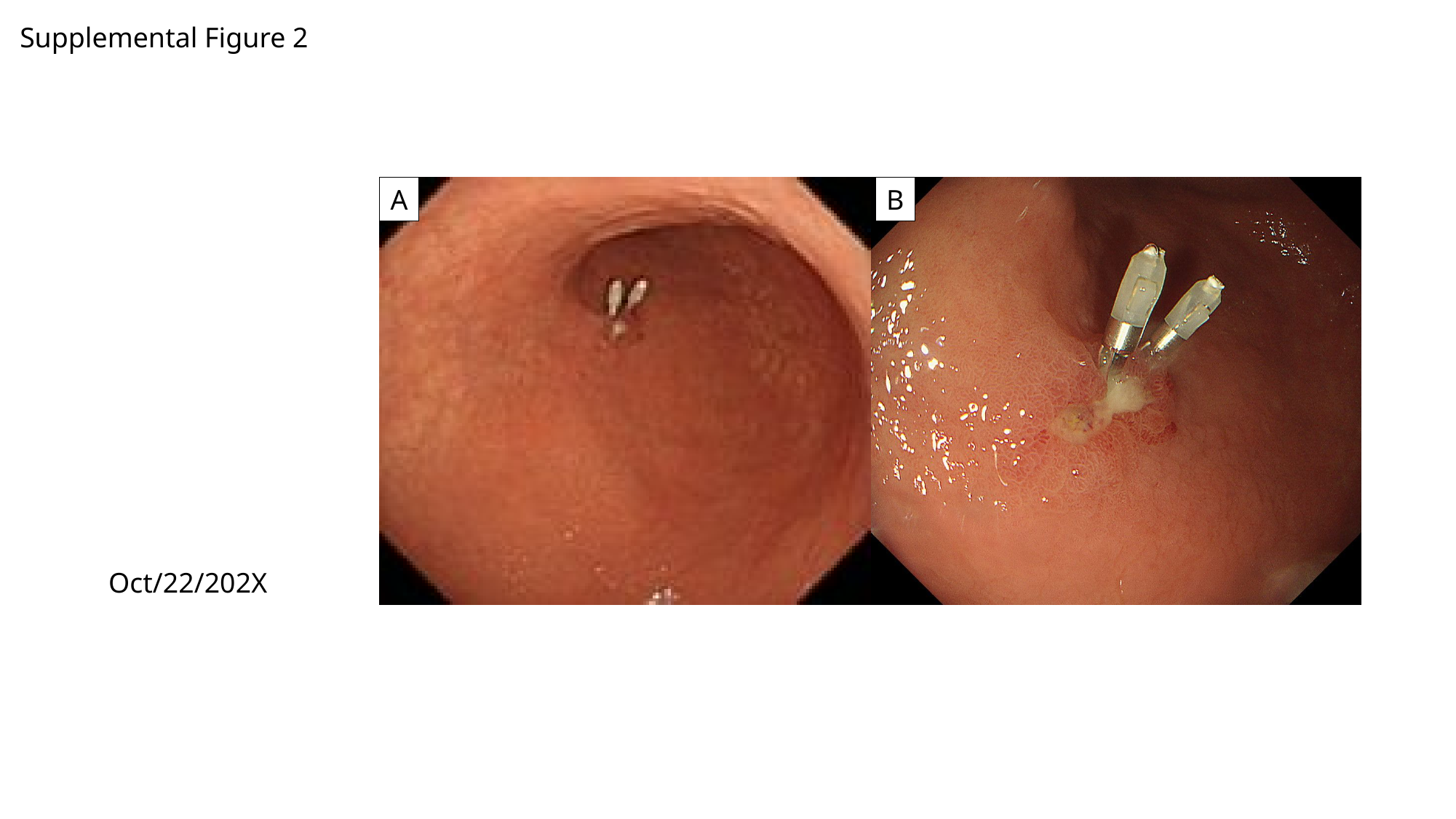

Supplemental Figure 2
A
B
Oct/22/202X

Supplement: Supplementary file 2 — FIGURE S2 (A) The clips remained in place at the time of follow‐up endoscopy 1 week later. (B) An ulcer base was observed at the root of the clips; however, no bleeding was identified. [file DEO2-7-e70337-s001.pptx]
